# L-Leucine increases the daily body temperature and affords thermotolerance in broiler chicks

**DOI:** 10.5713/ajas.18.0677

**Published:** 2018-10-29

**Authors:** Guofeng Han, Hui Yang, Yunhao Wang, Shogo Haraguchi, Takuro Miyazaki, Takashi Bungo, Kosuke Tashiro, Mitsuhiro Furuse, Vishwajit S. Chowdhury

**Affiliations:** 1 Graduate School of Bioresource and Bioenvironmental Sciences, Kyushu University, Fukuoka 819-0395, Japan; 2 Department of Biochemistry, Showa University School of Medicine, Tokyo 152-8555, Japan; 3 Department of Bioresource Science, Hiroshima University, Higashi-Hiroshima 739-8528, Japan; 4 Department of Molecular Biosciences, Faculty of Agriculture, Kyushu University, Fukuoka 819-0395, Japan; 5 Faculty of Arts and Science, Kyushu University, Fukuoka 819-0395, Japan

**Keywords:** L-leucine, Chicks, Heat Stress, Rectal Temperature, Thermotolerance

## Abstract

**Objective:**

Heat stress poses an increasing threat for poultry production. Some amino acids have been found to play critical roles in affording thermotolerance. Recently, it was found that *in ovo* administration of L-leucine (L-Leu) altered amino acid metabolism and afforded thermotolerance in heat-exposed broiler chicks.

**Methods:**

In this study, two doses (35 and 70 μmol/egg) of L-Leu were administered *in ovo* on embryonic day 7 to determine their effect on rectal temperature (RT), body weight (BW) and thyroid hormones at hatching. Changes in RT, BW, and thermotolerance in post-hatched chicks were also analyzed.

**Results:**

It was found that *in ovo* administration of L-Leu dose-dependently reduced RT and plasma thyroxine (T_4_) level just after hatching. In post-hatched neonatal broiler chicks, however, the higher dose of L-Leu administered *in ovo* significantly increased RT without affecting BW gain. In chicks that had been exposed to heat stress, the RT was significantly lowered by *in ovo* administration of L-Leu (high dose) in comparison with the control chicks under the same high ambient temperature (HT: 35°C±1°C, 120 min).

**Conclusion:**

*In ovo* administration of L-Leu in a high dose contributed to an increased daily body temperature and afforded thermotolerance under HT in neonatal broiler chicks.

## INTRODUCTION

Heat stress presents an increasing challenge for poultry production across the world since chickens are very sensitive to high ambient temperature (HT) [1]. The HT causes their body temperature to increase and induces oxidative stress [2,3]. Promoting the development of thermotolerance in chickens would therefore be one a timely approach to dealing with this challenge. Some free amino acids are recognized as biomarkers of heat stress, since they are significantly affected by heat stress [3,4]. These free amino acids play critical roles in thermoregulation, as well as in reducing body temperature in chicks under heat stress [5–9].

Leucine (Leu) has been demonstrated to play critical roles in physiological regulation, including in protein synthesis, in reducing muscle damage, and in glucose or lipid metabolic activity [10,11]. In our previous study, it was found that thermal manipulation (TM), which is a technique enabling the acquisition of thermotolerance in chickens [12], significantly decreased brain and hepatic Leu concentration in developing embryos [7]. *In ovo* administration of L-Leu (35 μmol/egg) on embryonic day (ED) 7 was then found to cause hypothermia at hatching, and also to afford thermotolerance in young broiler male chicks under HT [7,8]. However, the appropriate dose at which to administer L-Leu *in ovo* to develop thermoregulatory functions in broiler chicks is still unknown. The thyroid hormones thyroxine (T_4_) and triiodothyronine (T_3_) are associated with metabolic rate, which affects the regulation of body temperature [13,14]. It was concluded that TM during embryogenesis caused the thyroid and adrenal hormone levels to decline, resulting in hypothermia at hatching [15]. The first aim of this study was to compare the effects on body temperature and body weight (BW) of two doses (35 or 70 μmol/egg) of L-Leu administered *in ovo*, as well as their effects on thyroid hormone levels at hatching in broiler chicks.

Plasma triacylglycerol, non-esterified fatty acid (NEFA) and ketone body levels were significantly increased, and in addition, food intake significantly decreased, in male chicks administered with L-Leu *in ovo* compared with control chicks at 180 min of a heat challenge (35°C±1°C) [8]. The stimulated lipid metabolic activity was suggested as a possible endogenous factor contributing to the development of thermotolerance under heat stress in broiler chicks [8]. Therefore, it is important to examine the plasma metabolites. *In ovo* administration of a high dose of L-Leu could be expected to produce more potential effects of L-Leu in terms of affording thermotolerance in broilers and it could also help to clarify the mechanisms whereby L-Leu develops heat-resistant broilers. The second aim of this study was to investigate the effect of *in ovo* administration of a high dose (70 μmol/egg) of L-Leu on daily body temperature and BW, as well as on food intake, plasma metabolites and thermotolerance in chicks under heat stress.

## MATERIALS AND METHODS

### Experimental design

In Experiment 1, forty-five fertilized broiler eggs were purchased from a local hatchery (Mori Hatchery, Fukuoka, Japan) and placed in an incubator (SHOWA P008-type incubator, SHOWA Furanki Company, Saitama, Japan). The eggs were divided into three groups (n = 15/group) based on the initial egg weight, with the resulting groups being as uniform possible. The groups were as follows: control (sterile water injection in the amount of 500 μL/egg); L-Leu low dose (35 μmol/500 μL/egg); and L-Leu high dose (70 μmol/500 μL/egg) (average egg weight: control = 65.2 g; L-Leu (low) = 65.2 g; L-Leu (high) = 65.3 g). The eggs were incubated at a temperature of 37.6°C, at 58% to 68% relative humidity, and with auto-turning per h. The process of *in ovo* administration was the same as reported previously [7,8]. In brief, an injection was performed on ED 7. Unfertilized eggs were detected by candling and discarded prior to the injection. After sterilization, a small hole was made at the large end of the egg and sterile water or L-Leu solution was injected to a depth of 25 mm with a 1-mL disposable syringe that had a 25-gauge needle. The small holes were immediately sealed with Scotch tape after injection. At ED 18, the eggs were shifted to the hatching trays after detecting the undeveloped and dead embryos by candling and removing them. Rectal temperature (RT) and BW were recorded at 2 h after hatching. The RT was recorded by a digital thermometer with an accuracy of ±0.1°C (Thermalert TH-5, Physitemp Instruments Inc., Clifton, NJ, USA), by inserting the thermistor probe into the colon (rectum) through the cloaca to a depth of 2 cm, as we reported previously [7,16]. The chicks were properly anesthetized with isoflurane (Mylan Inc., Tokyo, Japan) before a blood sample was collected from the jugular vein. The blood was collected in heparinized tubes and centrifuged at 10,000×*g* at 4°C for 4 min in order to separate out the plasma.

In Experiment 2, fifty-four eggs were divided into two groups based on their initial weight. The experimental groups were: control (n = 27; average egg weight = 64.1 g) and L-Leu high dose (n = 27; average egg weight = 63.9 g). The incubation and *in ovo* administration processes were the same as described above in Experiment 1. After hatching, the chicks were housed in groups in metal cages (floor space: 36 cm×50 cm; height: 30 cm) under a control thermoneutral temperature (CT) with the following temperature schedule: 30°C for 1 to 4 days, and 28°C for 5 to 10 days. The chicks were provided with free access to food (Adjust diets; metabolizable energy >12.6 MJ/kg and crude protein >23%; Toyohashi Feed and Mills Co. Ltd., Aichi, Japan) and water as well as continuous light. In previous studies [7,8], *in ovo* administration of L-Leu was found to afford thermotolerance in young male broiler chicks. At 2 days old, therefore, the males were separated, by means of sexing through feather identification, for further experimental procedures to be carried out on them. The 19 males (control = 10; L-Leu high dose = 9) were subjected to a post-hatch experiment. At 8 days old, the males were individually isolated in small metal cages (floor space: 20 cm×28 cm; height: 30 cm). Since in our previous study the body temperature in birds administered with L-Leu *in ovo* was found to have significantly decreased 120 min after starting the heat stress during 180 min of heat exposure [8], in the current study we decided to set a time point of 120 min, when we would examine the effects of heat stress. At 10 days old, the male chicks from each group were randomly exposed to HT (35°C±1°C) for 120 min. The heat-exposed chicks were put into two chambers (Sanyo Electric Co. Ltd., Osaka, Japan; Catalog number: Sanyo MIR-254). All birds were provided with free access to food and water under HT. Food intake was measured on the basis of the amount of food that had disappeared over 120 min in the feeders. Adequate precautions were taken against food spillage – namely, the feeders were only half-filled so that chicks could not spill any food. RT was recorded at 0 min and 120 min of heat exposure. Plasma was collected following proper anesthetization with isoflurane (Mylan Inc., Japan), as in Experiment 1. All the collected samples were stored at −80°C until further analysis took place.

This study was performed in accordance with the guidelines for animal experiments in the Faculty of Agriculture of Kyushu University and complied with Law No. 105 and Notification No. 6 of the Japanese government.

### Analysis of plasma thyroid hormones

Plasma samples were subjected to LC-MS/MS analysis to determine T_3_ and T_4_ concentrations. Briefly, plasma samples were spiked with ^13^C_6_-T3 (T-087, Sigma, St. Louis, MO, USA) as an internal standard and were added to a mixture of 5% NH_4_OH and acetonitrile (50/50, [v/v]), and mixed thoroughly in a vortex. Samples were loaded onto SPE columns (EVOLUTE EXPRESS AX, Biotage, Charlotte, NC, USA) and extracted by using 5% formic acid in methanol. After evaporation, the samples were reconstituted in 2 mM ammonium acetate/0.1% formic acid/50% methanol for LC-MS/MS analysis. The samples were analyzed on a QTRAP 5500 LC-MS/MS system (AB SCIEX, Tokyo, Japan) connected to a Shimadzu LC 20A HPLC system. The multiple reaction monitoring transitions in positive ionization mode were as follows: for T_3_, 651.8>605.8; for T_4_, 777.8>731.8; for ^13^C_6_-T3, 657.9>611.9.

### Analysis of plasma metabolites

Plasma NEFA, ammonia (NH_3_), lactic acid (LA), lactate dehydrogenase (LDH), immunoglobulin A (IgA) and ketone bodies were examined using a reagent kit provided by the manufacturer to run in a Beckman Coulter AU480 automatic biochemistry analysis system (Beckman Coulter, Brea, CA, USA).

### Statistical analyses

The data concerning RT and relative BW, as well as thyroid hormones at hatching, were statistically analyzed by one-way analysis of variance (ANOVA) following a post-hoc analysis using Holm-Sidak’s multiple comparisons test that was carried out when a significant difference was detected. The data concerning daily RT and BW, as well as RT changes under heat stress, were statistically analyzed by a two-way repeated-measures ANOVA following a post-hoc analysis using Holm-Sidak’s multiple comparisons test that was carried out when a significant interaction was detected, where the main effects were *in ovo* administration of L-Leu and age or HT. The data concerning food intake under heat stress were statistically analyzed by *t*-test. Statistical analyses were performed using a commercially available package – GraphPad Prism 6 (GraphPad Software Inc., San Diego, CA, USA). Significant differences were denoted as p<0.05. Data were expressed as mean±standard error of the mean. All data in each group were first subjected to a Thompson’s rejection test to eliminate outliers (p<0.01) [17], and the remaining data were used for the analysis among groups. The number of chicks per group (n = 6–10), which was used in the current study generally considered as sufficient for statistical analysis, like one- or two-way ANOVA or *t*-test.

## RESULTS

### Rectal temperature, body weight and thyroid hormone concentrations at hatching

**Figure 1:**
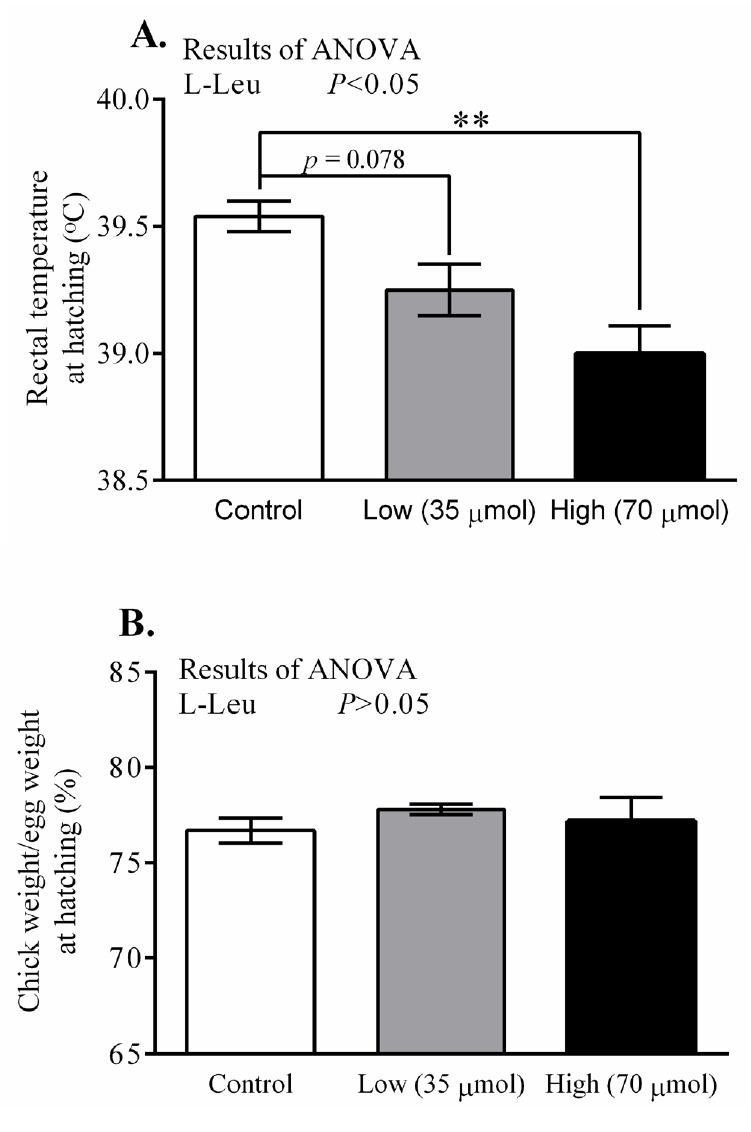
Effects of *in ovo* administration of L-Leu on rectal temperature (A) and the ratio of chick weight to egg weight (B) at hatching. The number of chicks in each group was as follows: control, n = 6; low, n = 10; high, n = 9. Values are expressed as mean±standard error of the mean. L-Leu, L-leucine; control, sterile water injection; low (35 μmol), L-Leu injection with 35 μmol/egg; high (70 μmol), L-Leu injection with 70 μmol/egg. ** p<0.01 by Holm-Sidak’s multiple comparisons test.

**Figure 2:**
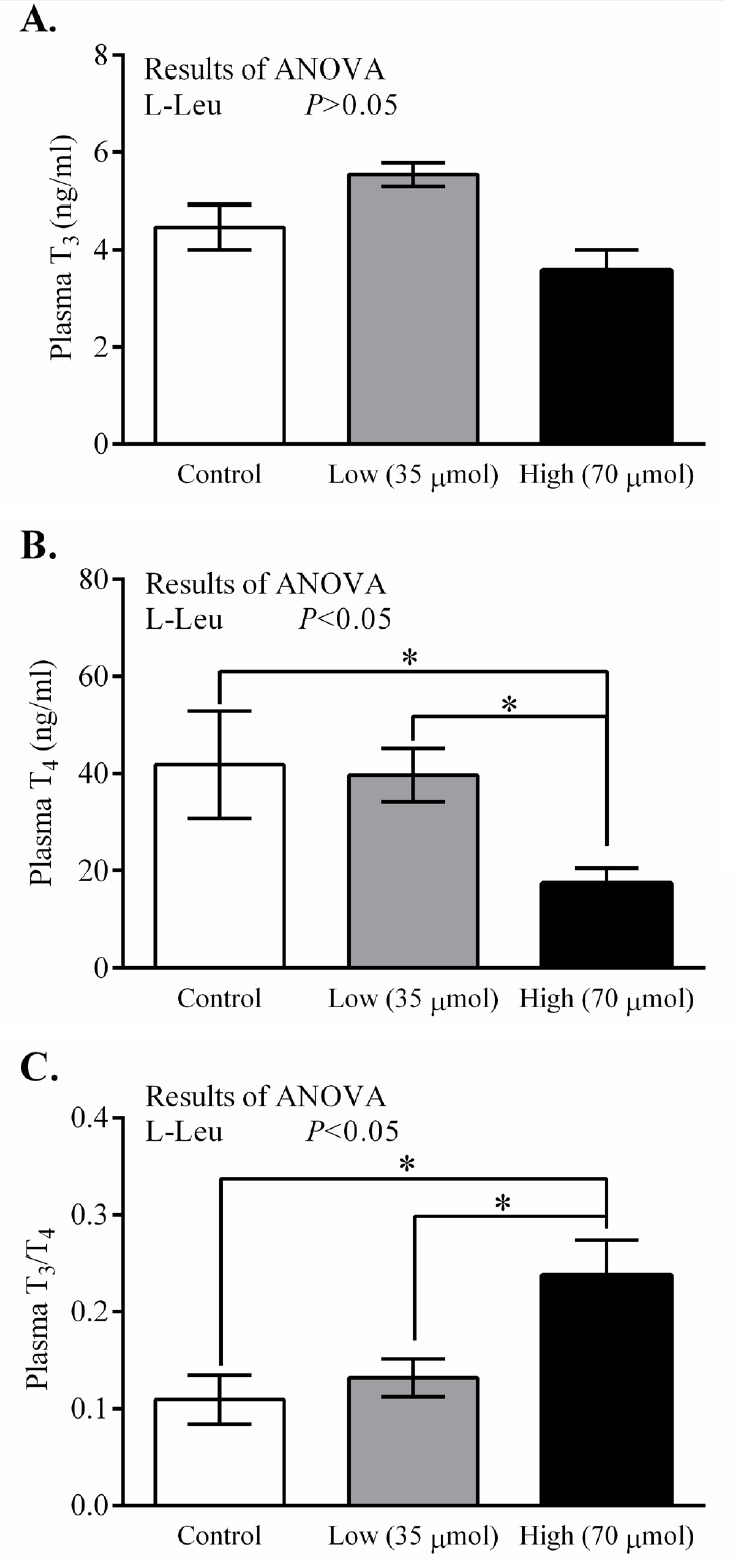
Effects of *in ovo* administration of L-Leu on plasma T_3_ (A), T_4_ (B), and T_3_/T_4_ ratio (C) at hatching. The number of chicks in each group was as follows: control, n = 6; low, n = 10; high, n = 9. Values are expressed as mean±standard error of the mean. L-Leu, L-leucine; control, sterile water injection; low (35 μmol), L-Leu injection with 35 μmol/egg; high (70 μmol), L-Leu injection with 70 μmol/egg. * p<0.05 by Holm-Sidak’s multiple comparisons test.

*In ovo* administration of L-Leu dose-dependently decreased (p<0.05) RT at hatching in comparison with control-administered chicks. RT was found to have significantly decreased in the high-dose L-Leu group, and it showed a tendency to decrease (p = 0.078) in the low-dose L-Leu group, compared with the control group (Figure 1A). However, *in ovo* administration of L-Leu did not affect the relative BW of chicks at hatching (Figure 1B). The thyroid hormone T_3_ was not affected by *in ovo* administration of L-Leu (Figure 2A). However, *in ovo* administration of a high dose of L-Leu significantly (p<0.05) reduced the plasma T_4_ concentration and increased (p<0.05) the ratio of T_3_/T_4_ compared with the control group and the L-Leu (low-dose) group (Figures 2B, 2C).

### Changes in rectal temperature and body weight under CT, as well as in RT, food intake and plasma metabolites under HT, in post-hatched broiler chicks

**Figure 3:**
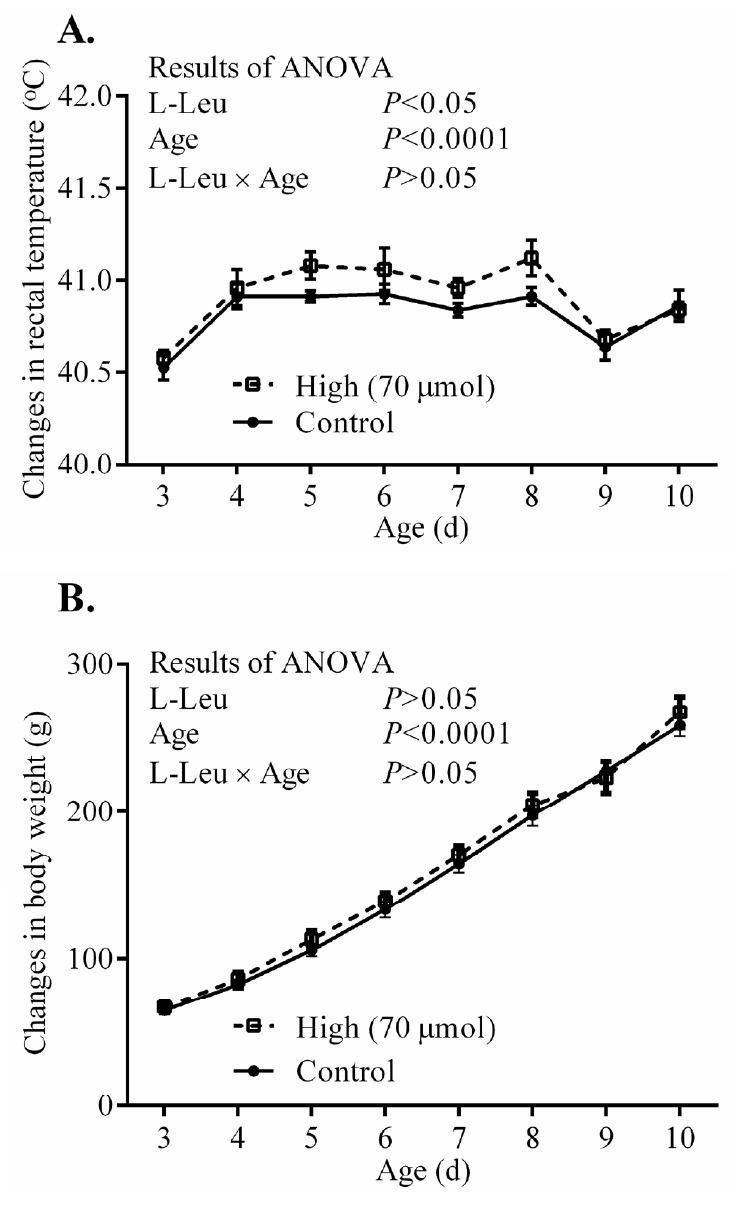
Effects of *in ovo* administration of L-Leu on changes in daily rectal temperature (A) and body weight (B) in post-hatched male broiler chicks. The number of chicks in each group was as follows: rectal temperature (control = 8; high = 6); body weight (control = 10; high = 8). Values are expressed as mean± standard error of the mean. L-Leu, L-leucine; control, sterile water injection; high (70 μmol), L-Leu injection with 70 μmol/egg.

**Figure 4:**
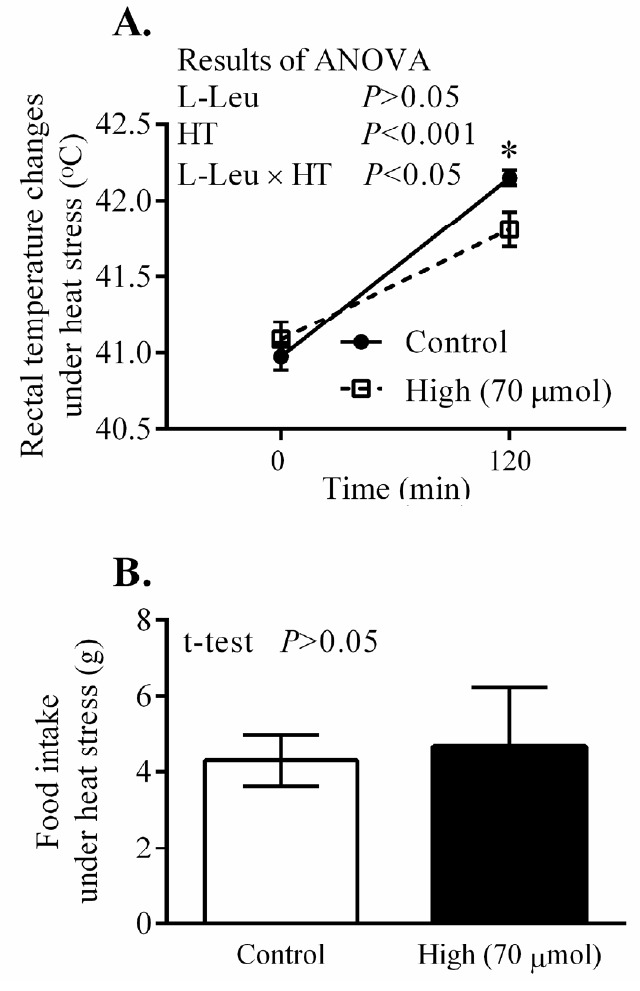
Effects of *in ovo* administration of L-Leu on changes in rectal temperature (A) and food intake (B) in heat-exposed male broiler chicks. The number of chicks in each group was n = 8. Values are expressed as mean±standard error of the mean. L-Leu, L-leucine; HT, high ambient temperature (35°C±1°C); control, sterile water injection; high (70 μmol), L-Leu injection with 70 μmol/egg.

The RT and BW significantly (p<0.0001) increased with age from 3 days old, but the values for RT were constant from 4 days old. *In ovo* administration of L-Leu significantly increased RT, but not BW gain, compared with the control treatment within the experimental days (Figure 3). HT caused RT to increase significantly (p<0.001) in broiler chicks. Notably, RT was significantly (p<0.05) lower in chicks administered with L-Leu *in ovo* than it was in control chicks administered *in ovo* under heat stress (Figure 4A), while food intake was not affected by *in ovo* administration of L-Leu under HT (Figure 4B). Plasma NEFA, NH_3_, LA, LDH, IgA, and ketone bodies were also not affected by *in ovo* administration of L-Leu in the current study (Table 1).

## DISCUSSION

In the current study, we administered two doses (35 and 70 μmol/egg) of L-Leu *in ovo* and examined their effects on RT, BW, and thyroid hormone levels at hatching in Experiment 1. The effects of high-dose administration of L-Leu (70 μmol/egg) on changes in daily RT and BW gain under CT, as well as on thermotolerance and food intake under HT, were examined in post-hatched chicks in Experiment 2. Accordingly, we confirmed that *in ovo* administration of a high dose of L-Leu has a stronger influence on regulation of body temperature at hatching. To the best of our knowledge, this is the first report to show that daily body temperature is higher in post-hatched chicks as a result of the *in ovo* administration of an amino acid—L-Leu. The plasma metabolites were also confirmed not to have contributed to the L-Leu-mediated improvement in thermotolerance.

Recently, we reported that *in ovo* administration of L-Leu (35 μmol/egg) once, on ED 7, has a strong influence in terms of stimulating embryonic metabolic activity and providing thermotolerance in post-hatched male broiler chicks under heat stress [7,8]. In the current study, males and females were not separated at hatching since sexing was conducted using the feather-identification method, which is only possible at 2 days old at the earliest. *In ovo* administration of L-Leu dose-dependently decreased RT at hatching. The tendency that was observed (p = 0.078) for RT to decline in the low-dose group compared with the control group might have been due to the sex-specific effects of *in ovo* administration of L-Leu, since it has been reported that females are more responsive to L-Leu administration in terms of showing a reduced RT at hatching [7]. At hatching, RT was clearly reduced in the high-dose group (p<0.01) compared with control chicks (Figure 1A). It has been reported that TM-dependent lower RT occurred at hatching due to a lower metabolic rate before hatching [18], which resulted from a feedback of higher metabolic rate under TM during embryogenesis [9]. Previously, we found that *in ovo* administration of L-Leu on ED 7 stimulated metabolic activity and lipid metabolism during the middle stages of embryogenesis [8]. Therefore, it could be hypothesized that the metabolic rate might be reduced before hatching due to the feedback of a higher metabolic rate in the middle of embryogenesis as a result of *in ovo* administration of L-Leu, which resulted in a lower RT at hatching in birds administered with L-Leu *in ovo*.

Thyroid hormones (T_4_ and T_3_) increase with the process of embryonic development and reach a peak before hatching [13,15]. Both T_4_ and T_3_ have been documented to stimulate the metabolic rate in birds, except at the neonatal stage, and to play a role in heat production as well as in adaptive variation in heat exposure [19,20]. In the current study, the plasma T_4_ concentration decreased in association with lower body temperature in the high-dose group in comparison with control birds at hatching, and this finding was similar to that in a previous study, where the TM-mediated thyroid hormone and body temperature were found to have declined at hatching [15,21]. It has been reported that the thyroid and the adrenal axis contribute to thermal regulation in neonatal chicks. Although intraperitoneal injection of T_3_ and T_4_ did not influence body temperature, the inhibition of the hypothalamus-pituitary-adrenal axis was found to lower body temperature [22]. Despite being homoeothermic creatures, neonatal chicks cannot keep their body temperature constant unless the environmental temperature is warmed [14]. This may be explained by the insufficient function of the thyroid hormones during the neonatal stage. TM during embryogenesis caused the function not only of the thyroid but also of the adrenal axis to decline, and resulted in hypothermia [12]. This hypothermia was mainly due to the reduced function of the adrenal axis [22]. Moreover, the function of T_4_ is weaker than that of T_3_ [14]. In addition, our recent studies showed that the concentration of plasma thyroid hormones (T_4_ and T_3_) was not affected by *in ovo* administration of L-Leu under short-term heat stress, and that even thermotolerance was improved in 4-week-old male broilers (unpublished data). Together, these findings suggest that the reduced body temperature found in the high-dose group at hatching might not be explained by the lower level of plasma T_4_.

After hatching, body temperature and BW gain increased with the progress of age in neonatal chicks [7,8,23]. Chicks, which are precocial birds, have a limited initial ability to maintain homeostasis of body temperature at hatching [14]; however, the thermoregulatory functions become well developed and are mature at 10 days old in broilers [24]. The higher level of body temperature in neonatal broiler chicks administered with L-Leu *in ovo* suggests that their thermoregulatory functions might have developed well or become mature earlier compared with control broilers. The higher body temperature might have been aided by the relatively high metabolic rate in chicks administered with L-Leu *in ovo*. However, the unaffected BW gain in chicks administered with L-Leu *in ovo* suggests that their food intake could be expected to have increased simply to support the high metabolic rate. Food intake and plasma thyroid hormones in post-hatched chicks need to be investigated in future studies.

Heat exposure significantly increased body temperature, which is a normal physiological response under heat stress [16]. Increased body temperature is beneficial for increasing sensible heat loss; however, it has been assumed that sensible heat loss does not play an important role when the ambient temperature is above the upper limit of the thermoneutral zone [25]. Prolonged heat exposure and high body temperature cause oxidative stress [3,26] in chickens. Body temperature is a reliable parameter of thermotolerance acquisition in avians [14]. Body temperature was significantly lowered in chicks administered with L-Leu *in ovo* compared with control chicks under heat exposure (Figure 4A), which suggests that *in ovo* administration of L-Leu improved the thermotolerance of chicks [7,8,27]. Higher heat loss or lower heat production, or both, contribute to lowering body temperature [28]. A previous study showed that food intake was reduced and lipid metabolism was activated after 180 min of heat exposure, and also that body temperature was significantly lower at 120 min of a heat challenge, in chicks administered with L-Leu *in ovo* [7,8]. In the current study, body temperature was significantly reduced at 120 min of heat stress in chicks administered with L-Leu *in ovo* in comparison with control birds; however, food intake and plasma metabolites (NEFA, ketone bodies) were not affected at 120 min of heat exposure (Figure 4B, Table 1). These results indicate that food intake and lipid metabolism might not be the main contributors to L-Leu-mediated improvement in thermotolerance. Our recent study also supports the finding that thermotolerance was improved without affecting food intake and lipid metabolism after 120 min of heat stress in 4-week-old broilers administered with L-Leu *in ovo* (unpublished data). Therefore, it could be speculated that *in ovo* administration of L-Leu might cause some epigenetic modulations in certain neural factors in broiler chicks to afford thermotolerance since the brain is the center of thermoregulation [14]. Future research will aim to clarify this issue.

In conclusion, the stronger effects of high-dose *in ovo* administration of L-Leu caused hypothermia and a reduced plasma T_4_ level at hatching, as well as enhancing the daily thermoregulation process in neonatal chicks and affording thermotolerance. The present study clarified that food-intake regulation and lipid metabolism did not contribute to the L-Leu-mediated improvement in thermotolerance under short-term heat stress. A future study will reveal the biochemical and molecular mechanisms whereby L-Leu improves thermotolerance.

## Figures and Tables

**Table 1:** Effects of *in ovo* administration of L-leucine on plasma metabolites in heat-exposed 10-days old male broiler chicks in Experiment 2

Items	LA (mg/dL)	LDH (mg/dL)	NEFA (mEq/L)	IgA (mg/dL)	NH_3_ (mEq/L)	Ketone body (mmol/L)
Control	45.5±3.3	560±39	319±42	18.5±0.11	225±7	404±38
High (70 μmol)	52.8±5.6	527±26	406±24	18.5±0.09	245±11	428±29
p-value	0.273	0.512	0.116	0.754	0.154	0.635

The number of chicks used in each group was 7 to 9. Values are means±standard error of the mean.

LA, lactic acid; LDH, lactate dehydrogenase; NEFA, non-esterified fatty acid; IgA, immunoglobulin A; NH_3_, ammonia; High (70 μmol), L-Leu injection with 70 μmol/egg.
